# Attitudes and usage of visual-aids in graduate student learning of gross anatomy at Makerere University

**DOI:** 10.4314/ahs.v23i1.67

**Published:** 2023-03

**Authors:** Peruth Nabirye, Lukiza B Paul, Erisa S Mwaka

**Affiliations:** 1 Department of Medical Illustration School of Biomedical Sciences, Makerere College of Health Sciences. P.O. Box 7072, Kampala, Uganda; 2 Department of Anatomy, School of Biomedical Sciences, Makerere College of Health Sciences. P.O. Box 7072, Kampala, Uganda

**Keywords:** Visual aids, usage, gross anatomy learning

## Abstract

**Background:**

The increasing numbers of students studying human anatomy at Makerere University are beginning to overwhelm available resources, which presents challenges in learning and necessitates an evaluation of alternative ways to enhance anatomy learning.The increasing numbers of students overwhelm resources available and presents challenges in learning. This necessitates an evaluation of alternatives to enhance gross anatomy learning at Makerere University College of Health Sciences. The study aimed to assess the usage of visual aids and generate necessary information to enhance learning.

**Methods:**

A cross-sectional study employing a concurrent triangulation mixed method design was conducted among 44 graduate students actively participating in cadaveric dissection. Data was collected using self-administered questionnaires and two focus group discussions. Descriptive statistics and thematic analysis were used to summarize data.

**Results:**

Most participants were male (77.3%) with a mean age of 31.5 years (SD 3.9, Range, 27-45 years). A majority of graduate students reported using non-traditional methods (online sources) to supplement learning. Students commonly used hard copies of Cunningham Manual of Practical Anatomy (88.6%) supplemented with well-illustrated textbooks (79.5%) and online dissection videos (72.7%). Students expressed most satisfaction with the use of videos and well-illustrated text books in supplementing learning. The availability of these electronic resources was limited by factors such as poor internet connectivity and the need to pay for online licenses and subscription fees.

**Conclusion:**

Graduate students in the Department of Anatomy at MakCHS are using non-traditional methods to supplement their learning. However, there are several challenges to accessing digital resources. There is a need to support students with accessing visual aids through integrating newer teaching modalities and modern technology to promote interest and retention of anatomical knowledge.

## Background

A critical review of the study about the best teaching practices in anatomy education emphasized that critical areas in learning gross anatomy such as full-body dissection would be best reserved for medical students with surgical career intentions^1^.

To effectively achieve learning objectives, students need the support of visual aids as multiple pedagogical resources in gross anatomy learning^2^. According to Burton, visual aids are sensory objects or images which motivate and support students learning^3^. They make the learning process more real, accurate, and active. Oyedele reports that teachers consider some visual aids as projected like motion pictures, slide film strips, demonstrations, enlargements and, non-projected visual aids like flannel boards, flip charts, models, real objects, overhead projectors, and pictorials (charts, photos, etc)^4^. However, the evolution of visual aids has brought into existence more digital resources in modern medical education. These have allowed a large volume of educational material to be easily presented and accessed electronically through the Internet including textbooks with images, and audio support, cadaveric dissection videos, animated pictures, and three-dimensional visual aids such as virtual cadavers, anatomage table, radiology workstations, ultrasound sessions, and volumetric reconstruction^5^.

Technological advancement has also influenced students' growing preference for visualising moving images with audio explanations as opposed to older traditional static resources, such as text and still pictures^6^. Students at the Peninsula College of Medicine and Dentistry in the United Kingdom found it beneficial to gain anatomical knowledge from anatomy videos on YouTube and virtual models^7^,^8^. However, some teachers of anatomy are not convinced with the new developments of pedagogical tools that have substituted dissection in gross anatomy learning^9^. Research suggests that consolidating online libraries with the; traditional library system, dissection laboratories, and classrooms can create a versatile unified system of information^10^. This grants student access to a variety of materials, in different formats such as data, texts, images, and other forms of information both in print and web-based information. Although the internet and instructional technology provide reliable information and improve access to visual aids^5^, there is a challenge of unreliable internet connectivity^11^. This discourages students and affects access to multi-media services and online academic facilities^11^ which poses challenges in gross anatomy learning especially with the increase in student numbers versus the resources available. This study, therefore, assessed the usage of visual aids in graduate learning of gross anatomy to contribute to enhancing student learning of anatomy at MakCHS.

## Methodology

This was a cross-sectional study that employed a concurrent triangulation mixed method design 12 to obtain different but complementary data on the use of visual aids in learning gross anatomy in the Department of Anatomy at Makerere University College of Health Sciences in Uganda. The study population comprised 44 graduate students of human anatomy, psychiatry, and several surgical disciplines including general surgery, orthopedic surgery, otorhinolaryngology, and maxillofacial surgery. Only students actively participating in gross-anatomical cadaveric dissection were eligible for this study. Data was collected using a self-administered questionnaire with close-ended questions and two focus group discussions (FGD). The questionnaire was adapted from two sources^13^ and 14. The data collected included socio-demographic characteristics; knowledge, usage, and preference of visual aids in learning gross anatomy; challenges faced while using the various visual aids; and the sources of the visual aids. Perceptions on the use of visual aids were assessed by asking participants to give their level of satisfaction with the various available visual aids on a 5-point Likert scale ranging from very unsatisfying to very satisfying. Two FGDs involving 13 graduate students were also conducted to obtain deeper understanding of students' experiences in regard to the use of visual aids for learning gross anatomy.

All graduate students actively participating in full-body cadaveric dissection in the Department of Human Anatomy were eligible for the study and 44/45 were recruited for the survey. Participants for the FGD were purposively selected because of their keen interest in full body cadaveric dissection. Data were collected using a FGD guide that was developed by the authors and piloted on three graduate students who were excluded from the study. Participants were directly contacted and convenient appointments made. Both FGDs were conducted in the Department of Anatomy at MakCHS, audio recorded and later transcribed verbatim. Field notes were also taken during the interviews. Debriefing meetings were held by the research team at the end of each FGD to agree on the different perspectives that had been taken. The FGDs were facilitated by the first author (PN) assisted by one research assistant who also was the note-taker. Both FGDs were audio-recorded with the consent of all participants. On average, the FGDs lasted for 45 minutes.

### Data analysis

All questionnaires were immediately checked for completeness, cleaned and exported to SPSS Version 16.0 for analysis. Descriptive statistics were used to summarise the data.

Two of the authors (PN and EM) developed a codebook and coding framework based on two thematic areas; disagreements were solved by consensus. Transcripts were manually coded by two researchers (PN and EM). Data analysis and interpretation was conducted continuously throughout the study by all authors using a thematic approach^15^, ^16^. The first step of the analysis involved reading of all transcripts to familiarize, mark and memo the data. We then performed open line-by-line coding to generate the first set of codes. The codebook was then refined to identify themes in relation to participants' experiences in the use of visual aids for learning gross anatomy. Themes were supported by representative quotes.

Ethics approval was obtained from Makerere University School of Biomedical Sciences Research and Ethics Committee (SBS-HDREC – 674). Written informed consent was obtained from participants before participation in the study. All participants were assured of confidentiality.

## Results

The mean age of participants was 31.5 (SD 3.9, Range, 27-45 years) and the majority were male (34/44, 77.3%). Almost half of the participants (21/44, 47.7%) were graduate students pursuing a master of medicine degree in general surgery (Table 1).

**Table 1:** Academic programs of graduate students (n= 44)

Academic program	Freq. (%)
General surgery	21 (47.7)
Orthopedic surgery	10 (22.7)
Human anatomy	4 (9.1)
Ear-Nose and Throat surgery	3 (6.8)
Maxillofacial surgery	3 (6.8)
Psychiatry	3 (6.8)

Most students (93.2%) had used visual aids for learning gross anatomy in the dissection laboratory and; reported using both electronic and traditional visual aids. The most commonly used visual aid was Cunningham's dissection guide hard copy (39/44,88.6%) and textbook illustrations as shown in Table 2. Among the electronic visual aids, online dissection videos (32/44, 72.2 %) and downloaded PowerPoint presentations (28/44,63.6%) were the most commonly used.

**Table 2:** Visual aids used by graduate students in the gross anatomy laboratory (n= 44)

Electronic Visual aids	Frequency (n/%)
Dissection videos	32 (72.7%)
Digital posters	11 (25%)
Photographs	20 (45.4%)
Animations	8 (18.1%)
Radiological images	17 (38.6%)
Power Point presentation	28 (63.6%)
YouTube videos	7 (15.9%)
**Traditional visual aids**	
Textbooks with illustrations	35 (79.5%)
Dissection Manuals (Cunningham)	39 (88.6%)
Blackboards	31 (70.4%)
Charts	6 (13.6%)
Models	21 (47.7%)
**Source of visual aids**	
Internet	40 (90.9%)
Library	23 (52.2%),
Lecturers	21 (47.7%)
Anato-media	6 (13.6%)
Radio-pedia	1(2.2%)

Almost all participants obtained their visual aids by browsing the Internet (40/44, 90.9%). However, several (25/44, 56.8%) complained of poor Internet connectivity. The other challenges reported included: the inability to pay for online licenses and subscriptions (23/44, 52.2%); some videos are in foreign languages that participants cannot understand (17/44, 38.6%); lack of familiarity with smart devices (5/44, 11.3%); and lack of access to a computer and/or smartphones (3/44, 6.8%).

On the other hand, students expressed dissatisfaction with the contribution of digital posters to the understanding of gross anatomy. Only 2.2% of the students reported that posters enable them to recall tough points and facilitate better clinical correlation, 6.8% also reported that digital posters facilitate in understanding a large number of facts and clarity of concepts as well as effective utilization of time.

### Student's satisfaction with the use of visual aids for learning gross anatomy

The findings from the FGDs enabled us gain a deeper understanding of graduate students' experiences with the use of the various visual aids in learning gross anatomy. Most students reported being satisfied with the use of videos. They opined that videos facilitate effective utilization of time and enable them better understand difficult concepts.

*“...we have found learning easy because before lectures we watch videos on YouTube, it opens up the mind and enables us to appreciate the different approaches to understanding the content. When we go for the lecture, it is easy to absorb whatever people are presenting and by that, we can accumulate more time to discuss areas that we identified as complicated ...”.* (B, FDG 1).

Graduate students also acknowledged that videos supplement different teaching methods during learning. This was clearly expressed by a participant who stated

*“... Considering that we learn through so many ways, some of us are visual learners, who benefit a lot from videos because they are more elaborate., for example, teachers, or discussants share information through, classroom discussion, dissection, demonstration, seminars, and many other ways, so as one is able to learn from the presentation being made, they also learn from a video which demonstrates key concepts. So, I find videos and textbooks very important.* (C, FDG 2).

Much as a majority of graduate students expressed preference for videos as a learning aid, they noted that many of the informative videos are not freely accessible

*“...there are some very good video sites or websites that are recommended for university students to use. However, we cannot readily get access to them because we have to pay for the subscription charges yet we do not have money. The university is supposed to incur those costs but it assumes that the mere fact that it provided the internet then, it provided access to these websites...”.* (E, FGD1)

In addition to videos, graduate students also expressed the desire for visual aids that give a three-dimensional approach to gross anatomy. They argued that such visual aids are easy to manoeuvre and provide a multidimensional aspect to learning gross anatomy.

*“...Some of the visual aids are in 2D form, so you may not get a proper understanding of what you exactly want, because along the way you might get lost. However, some 3D visual aids enable you to manoeuvre and rotate especially those programs where you can dissect and, rotate in all angles...”.* (B, FGD 2)

Graduate students also suggested that the institution (MakCHS) should provide students with anatomical models

*“...since models are few; I think it would be good if the university invests in the different models of body parts. There are some sites where you find elaborative models that can clearly show a picture in gross anatomy learning. You do not have to over struggle...”.* (E FGD 1).

## Discussion

Visual aids are very vital in learning gross anatomy. Findings from this study suggest that a vast majority of graduate students in the Department of Anatomy at MakCHS are using non-traditional methods (available via the internet) to supplement their learning of gross anatomy however the availability of these electronic resources is limited by factors such as poor internet connectivity and the need to pay for online licenses and subscription fees. Among the available visual aids, videos were seen as the most preferred because they were perceived as good for effective utilization of time and enabled them better understand difficult concepts.

Similar to our findings, a critical review of the best teaching practices in anatomy education suggested that the best way to teach modern anatomy is combining multiple pedagogical resources, with emphasis that full-body dissection be best reserved for students with surgical career intentions 1. It is important to recognise that learning anatomy requires significant amounts of visual identification especially for purposes of safe clinical procedures such as in surgery. This is of utmost importance to our study participants since a greater majority (84%) were surgical residents. Understanding the spatial relationships and visual representations of anatomy structures is a good practice long-lasting understanding of the subject. The evolution of visual aids has generated both traditional and electronic visual aids that were being used by graduate students during learning. For better comprehension, students reported supplementing traditional dissection manuals (Cunningham's manuals of practical anatomy) with videos although albeit with some challenges. Students suggested that MakCHS as an institution should provide free access to these resources including physical anatomical models.

Almost all participants in this study obtained their visual aids from the Internet although they reported challenges such as poor Internet connectivity, inability to pay for online licenses and subscription fees, language barrier in some videos, lack of familiarity with smart devices and lack of access to a computer and/or smartphones. This concurs with various studies that have reported low internet bandwidth that as a hindrance to adequate utilization of online resources for learning in most institutions of higher learning across Africa 11, 17. The future of anatomy teaching will depend more on visual aids outside the dissection room because of increased access to web-based computer-aided instruction resources that support student learning 18. In the past few years there has been a sharp increase in student intake at MakCHS without concomitant increase in the available teaching resources, including cadavers. This state of affairs has created an imbalance that has inadvertently forced students to search for alternative sources of information such as e-learning, and with good results 19.

Findings from the FGD helped us better understand participants' preferences and the reasons for their satisfaction of the different visual aids they reported using in augmenting their learning of gross anatomy. A majority of graduate students reported satisfaction with the use of visual aids, particularly videos, because the perceived them as providing a multi-dimensional approach to learning. They further asserted that for better understanding of gross anatomy, videos should preferably be used before attending lectures and in preparation for cadaveric dissection. Studies show that videos improve student performance in gross anatomy and students are satisfied in using them for better comprehension of difficult concepts^20^–^22^.

Students in this study expressed the desire for three-dimensional (3D) visual aids because they provide a multi-dimensional approach to gross anatomy and enables them to appreciate structures from all angles. This finding is consistent with literature that shows preference for 3D visual aids in learning 23–25. The use of 3D tools has been shown to result in higher factual and spatial anatomy knowledge compared to traditional methods 25.

## Study limitations

The major limitation of this study was the small sample size and the lack of diversity in research participants. It would have been better to seek the opinions of anatomy teachers, librarians and medical illustrators who are key in ensuring that students adequately understand during learning. This was due to the limited timeframe and resources since this work was self-funded by the first author (PN) and it was conducted in partial fulfilment for a master of science in Medical Illustration. This also affected the generalizability of our findings however; this has laid ground for further research in this important field of maximizing comprehension in medical education.

## Conclusion

Our findings suggest that a vast majority of graduate students in the Department of Anatomy at MakCHS are using non-traditional methods (available via the internet) to supplement their learning of gross anatomy however, the availability of these electronic resources is limited by factors such as poor internet connectivity and the need to pay for online licenses and subscription fees. Most students expressed most satisfaction with the use of videos and well-illustrated text books in supplementing the traditional methods of learning gross anatomy. There is a need to guide and support students with accessing visual aids through integrating newer teaching modalities and modern technology to encourage interest and retention of anatomical knowledge, its clinical relevance and also address challenges of shortage of visual aids and satisfaction to make learning objectives more effective.

## Figures and Tables

**Table 3:** Satisfaction with the use of visual aids

Visual aids	Satisfaction with the use of visual aids					
	Very unsatisfying Freq (%)	Not satisfying Freq (%)	Fairly satisfying Freq (%)	Satisfying Freq (%)	Very satisfying Freq (%)	No response
Blackboards	8 (18.1)	7 (15.9)	15 (34.0)	9 (20.4)	1 (2.2)	4
Videos	1 (2.2)	0	4 (9.0)	11 (25)	25 (56.8)	3
Textbooks	0	1 (2.2)	12 (27.2)	15 (34.0)	13 (29.5)	3
Posters	8 (18.1)	5 (11.3 )	17 (38.6)	5 (11.3)	2 (4.5)	7
Combined visual aids	3 (6.8)	0	2 (4.5)	6 (13.6)	27 (61.3)	38
		**Visual aids**				
Reason for satisfaction		Blackboards Freq (%)	Textbooks Freq (%)	Videos Freq (%)	Digital Posters Freq (%)	
Recall tough points		11(25)	4(9.0)	21(47.7)	1(2.2)	
Better clinical correlation		4(9.0)	24(54.5)	10(22.7 )	1(2.2)	
Effective utilization of time		4(9.0)	6(13.6)	21(47.7)	3(6.8)	
A large number of facts and clarity of concepts		6(13.6)	20(45.4)	7(15.9)	3(6.8)	
Understand anatomy diagrams easily		8(18.1)	12(17.2)	15(34.0)	11(25)	
